# Mammalian SRP receptor switches the Sec61 translocase from Sec62 to SRP-dependent translocation

**DOI:** 10.1038/ncomms10133

**Published:** 2015-12-04

**Authors:** Bhalchandra Jadhav, Michael McKenna, Nicholas Johnson, Stephen High, Irmgard Sinning, Martin R. Pool

**Affiliations:** 1 BZH, University of Heidelberg, Im Neuenheimer Feld 328, D-69120 Heidelberg, Germany; 2 Faculty of Life Sciences, University of Manchester, Michael Smith Building, Oxford Road, Manchester M13 9PT, UK

## Abstract

Two distinct pathways deliver secretory proteins to the Sec61 protein translocase in the endoplasmic reticulum membrane. The canonical pathway requires the signal recognition particle (SRP) and its cognate receptor (SR), and targets ribosome-associated proteins to the Sec translocase. The SRP-independent pathway requires the Sec translocase-associated ER membrane protein Sec62 and can be uncoupled from translation. Here we show that SR switches translocons to SRP-dependent translocation by displacing Sec62. This activity localizes to the charged linker region between the longin and GTPase domains of SRα. Using truncation variants, crosslinking and translocation assays reveals two elements with distinct functions as follows: one rearranges the translocon, displacing Sec62 from Sec61. A second promotes ribosome binding and is conserved between all eukaryotes. These specific regions in SRα reprogramme the Sec translocon and facilitate recruitment of ribosome-nascent chain complexes. Overall, our study identifies an important function of SR, which mechanistically links two seemingly independent modes of translocation.

## Abstract (web summary)

Sec62 is a membrane-bound protein that is involved in the translocation of proteins via the signal recognition particle-independent pathway. Here, the authors show that the receptor SRα displaces Sec62 from the translocon and isolate the domain on SRα that is responsible for this.

## Graphical abstract

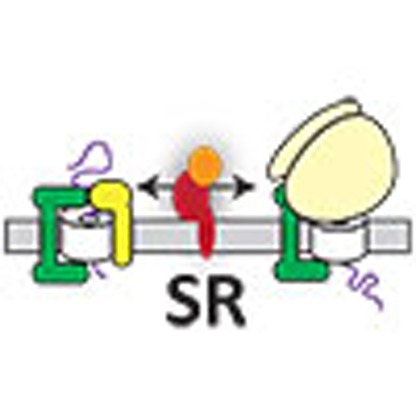

Proteins destined for secretion from the cell typically possess an N-terminal hydrophobic signal sequence, which targets them to the protein-translocating channel at the endoplasmic reticulum (ER) membrane^1^. Once the protein has been translocated across the membrane, the signal sequence is usually cleaved off and the mature protein can then fold and move through the secretory pathway via vesicular trafficking. Several distinct targeting pathways deliver proteins to the protein-conducting channel, which is formed by the heterotrimeric Sec61 complex (Sec61αβγ)^2^. Perhaps the best-characterized pathway is the canonical signal recognition particle (SRP) pathway, which is conserved in all domains of life^3^,^4^. SRP is a ribonucleoprotein complex, which binds to ribosomes and can recognize the signal sequence as it emerges from the ribosome exit tunnel^5^,^6^. Recognition of the signal sequence by the SRP54 component of SRP leads to a transient retardation in translation concomitant with targeting to the ER membrane via an interaction with the cognate SRP receptor (SR)^7^,^8^,^9^,^10^. SR mediates the transfer of the ribosome, together with the nascent chain, to the Sec61 translocase^11^. Translation resumes and the nascent chain is fed directly from the ribosome into the pore of the Sec61 translocon, which is co-aligned with the ribosome exit tunnel^12^. SRP then can be released from its receptor such that both can now participate in further rounds of targeting^13^.

In eukaryotes, SR is composed of two subunits, the 70 kDa peripheral membrane protein SRα (ref. 14), which is anchored to the membrane by the 30 kDa SRβ, which has a single N-terminal transmembrane (TM) domain and short luminal domain^15^. Both SRα and SRβ are GTPases; SRα has a bacterial homologue FtsY, and both of them share a characteristic GTPase domain (NG domain), which is also found in SRP54 and its bacterial homologue Ffh, as well as in a third member of the SRP GTPases, FlhF^16^,^17^. The NG domains of SRα and SRP54 interact in a GTP-dependent manner. Complex formation is kinetically accelerated by the presence of the SRP RNA and by the binding of a *bona fide* signal sequence^18^,^19^. Studies with the bacterial SRP–SR complex reveal that its subsequent interaction with the translocon (SecYEG) induces molecular rearrangements, which stimulate GTP hydrolysis in both GTPases leading to the release of signal sequence from SRP and its transfer to the translocon^20^.

SRα is bound to SRβ by its SRX domain, which possess a longin domain fold^21^,^22^. The NG and SRX domains are connected by a flexible linker, rich in charged residues, whose function is poorly characterized. FtsY lacks such linker and SRX domains, and instead possesses a natively unfolded A-domain, which is important for membrane binding via two lipid-binding motifs^23^,^24^,^25^. SRβ, in contrast, is closely related to the ARF and Sar1 family of GTPases^15^,^26^. However, details of the SRβ GTPase cycle are not well understood.

Not all secretory proteins use the SRP targeting pathway; characterized first in the yeast *Saccharomyces cerevisiae*, an SRP-independent translocation pathway was identified that, instead of SRP, absolutely requires the ER membrane protein Sec62 (ref. 27). Sec62 associates with the same core Sec61 channel used by the SRP pathway, forming a larger Sec translocase, alongside Sec63, Sec71 and Sec72 (ref. 28). Furthermore, there is no obligate coupling of translation and targeting with this pathway, unlike with SRP-dependent targeting. Substrates can be released from the ribosome and maintained in an unfolded conformation by chaperones of the heat-shock protein 70 (Hsp70) family^29^,^30^. They can then interact with the Sec translocase, which again forms the protein-conducting channel. In this case, the action of the ER Hsp70s (Kar2 and Lhs1), which are recruited to the luminal face of the Sec translocase by Sec63, drive the energetics of the translocation reaction^31^,^32^.

The pathway a particular yeast precursor will take is largely determined by the signal sequence, with more hydrophobic signal sequences being targeted via SRP and less hydrophobic sequences translocated via the Sec62 pathway^33^. The Sec62 pathway is, however, not restricted to yeast; homologues of Sec62 and Sec63, but not the non-essential Sec71 or 72, are also present in higher eukaryotes and furthermore can complement deletions of their homologues in yeast; suggesting that they perform similar functions^34^,^35^,^36^.

Recently, the first substrates for the mammalian Sec62 pathway were identified and they are typically short secretory proteins including insulin, apelin and statherin^37^,^38^,^39^, as well as the insect protein preprocecropin, which was known for a long time to translocate independently of SRP^40^. The fact that these precursors are very short makes them poor substrates for SRP, as they are likely released from the ribosome before SRP has had a chance to bind to their signal sequence efficiently. Before binding to the translocon, some of these short secretory proteins can also interact with upstream cytosolic factors, including calmodulin and components of GET-targeting pathway, which typically delivers C-terminally anchored membrane proteins to the ER^39^,^41^,^42^.

Both the SRP- and Sec62-dependent pathways converge at the Sec61 translocase, but require different accessory factors to associate with Sec61. SR and ribosomes are required in the SRP pathway, and Sec62 in the SRP-independent Sec62 pathway. Evidence suggests that Sec63 is likely to be involved with both pathways^43^,^44^. At present, little is known about the dynamics and interconvertability of these complexes.

Here we have explored the interaction of both Sec62 and SR with the mammalian translocon and find that interaction of ribosomes and Sec62 with the core Sec61 translocon is mutually exclusive. Furthermore, we identify a specific role for SR, involving the SRα linker domain, in displacement of Sec62, which provides a mechanism for the interconversion of translocons.

## Results

### Sec62 interacts with Sec61 in a ribosome-sensitive manner

**Figure 1:**
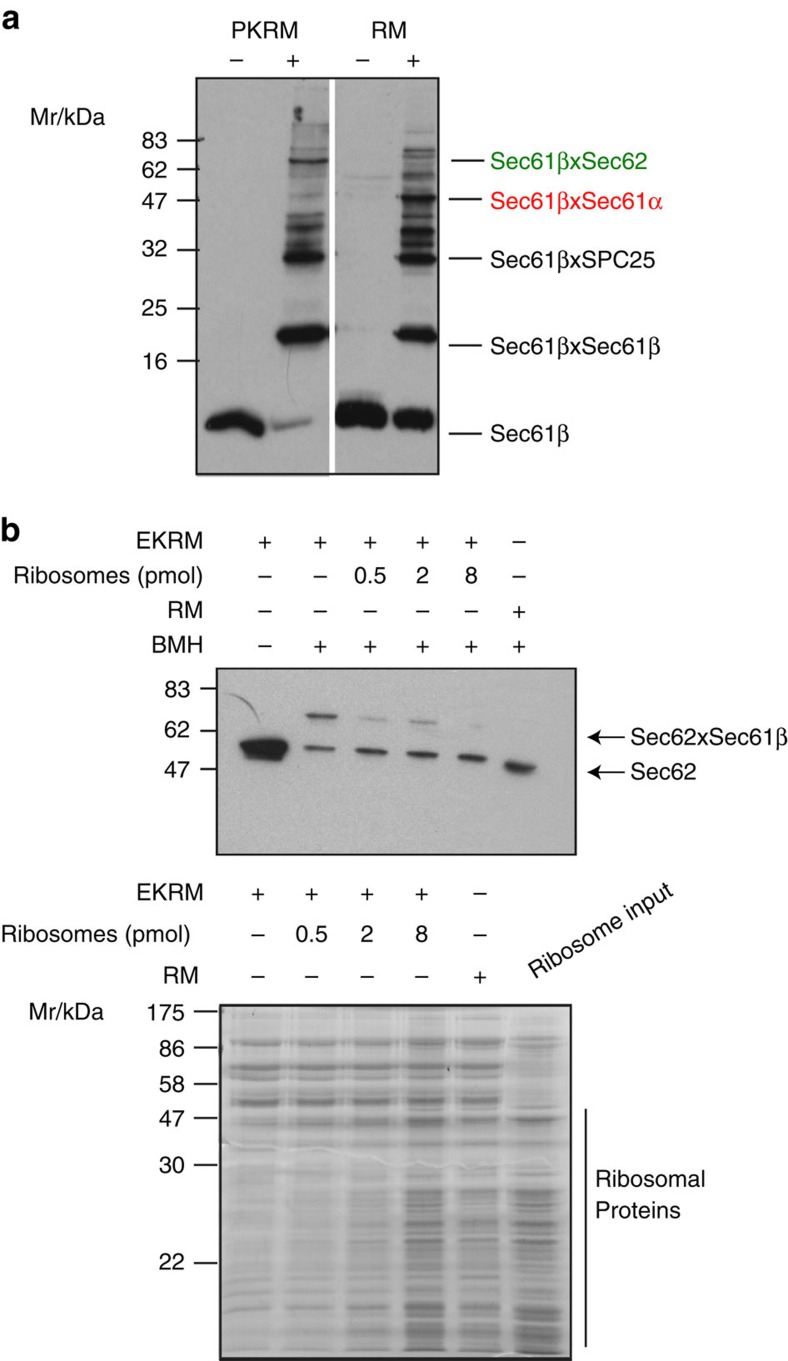
Sec62 interacts with the Sec61 complex in a ribosome-sensitive manner. (**a**) Equivalent amounts of RM and PKRM (10 eq) were treated with either DMSO or BMH (10 μM) and then analysed by SDS–PAGE and western blot with anst-Sec61β antisera. Positions of major cross-link species are indicated. (**b**) EKRM (8 eq) were incubated with increasing amounts of purified high salt-washed canine ribosomes (as indicated) before crosslinking as in **a**. An equivalent amount of RM were treated similarly with BMH. Samples were then analysed by western blot with anti-Sec62 antibodies. The position of the major Sec62 × Sec61β cross-link species is indicated. Binding of ribosomes to EKRM was monitored in parallel by membrane-pelleting and analysis of the pellet fractions by SDS–PAGE and staining with Coomassie Brilliant Blue.

We first compared the interaction profile of Sec61 with translocon-associated proteins in intact canine pancreatic rough microsomes (RMs), where >80% of translocons are engaged with ribosomes^45^, and microsomes stripped of ribosomes by puromycin-high salt treatment (PKRMs). The compound bismaleimidohexane (BMH), a cysteine-reactive homobifunctional reagent was used to monitor crosslinking from the single endogenous cysteine in Sec61β (Fig. 1a). As reported previously^46^, the Sec61β cross-link profile was strikingly different for these two preparations. In particular, crosslinking between Sec61β and Sec62 was very weak in the RM, but dramatically increased in the PKRM.

We repeated this experiment with membranes stripped of ribosomes with EDTA and high salt (EKRM) and monitored crosslinking of Sec62 by western blot. As observed with PKRMs, there was a dramatic enhancement in Sec62–Sec61β crosslinking with EKRMs compared with RMs (Fig. 1b). In fact more than 50% of Sec62 could be cross-linked to Sec61β with EKRMs (Fig. 1b). To test whether the loss of crosslinking between these two components was specifically due to the removal of ribosomes from the membrane, purified canine salt-washed ribosomes were allowed to rebind to the EKRMs and the cross-link assay was repeated (Fig. 1b). This led to a dose-dependent reduction in crosslinking between Sec62 and Sec61β. Hence, as reported previously^34^, the Sec62–Sec61β interaction is strongly dependent on the absence of ribosomes.

### Binding of SR to Sec61 positions SRα close to Sec61β

**Figure 2:**
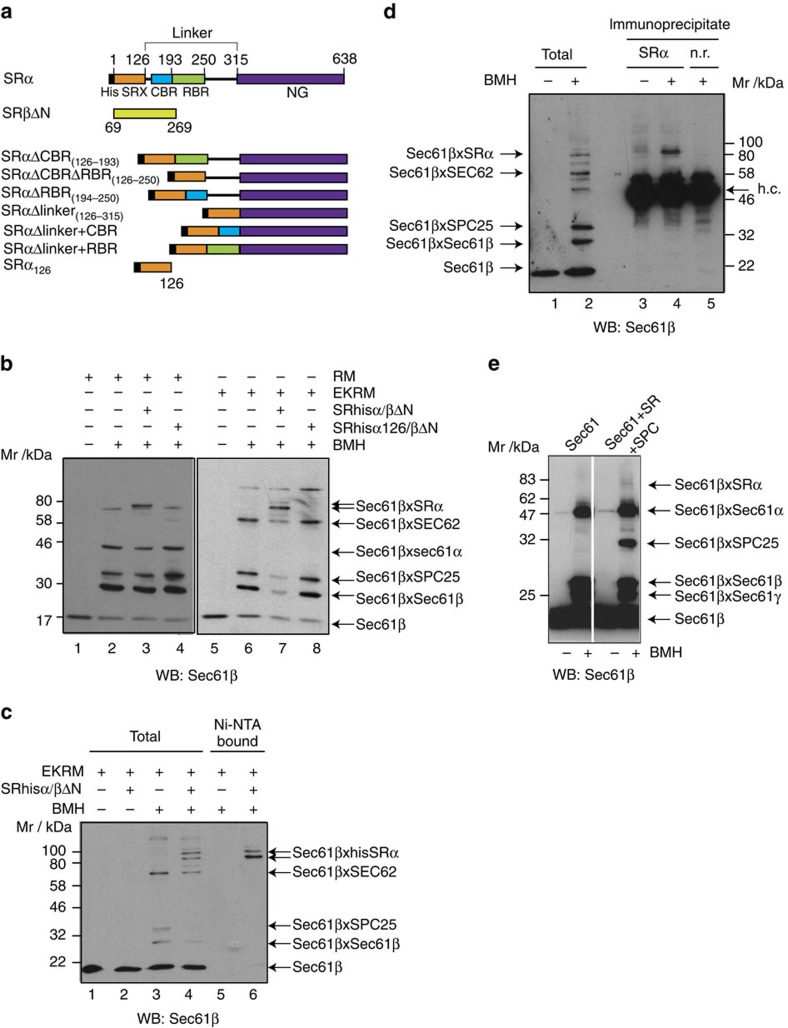
SR binds to the translocon in close proximity to Sec61β. (**a**) An overview of the SR constructs used in this study. (**b**) RM or EKRM (32 eq) were preincubated alone or with purified recombinant SRP receptor (7.5 μM), SRα/βΔN or a mutant SR, lacking the entire linker and NG domain of SRα (SRα_126_/βΔN). Samples were then analysed by BMH crosslinking followed by SDS–PAGE and western blot with Sec61β antibodies. (**c**) EKRM preincubated with and without SRα/βΔN, were treated, where indicated, with BMH (10 μM). Samples were then solubilized with Triton X-100 and high salt and then incubated with Ni-NTA beads to recover hisSRα together with any cross-link products that also contain hisSRα. Samples were then analysed by SDS–PAGE and western blot with Sec61β antibodies. (**d**) EKRM were treated with BMH (40 μM) before denaturing immuno-precipitation with either anti-SRα or a non-related antiserum (n.r.). An aliquot of the total reaction (5%) along with the immunoprecipitated material was analysed by SDS–PAGE and western blot with Sec61β antibodies. Positions of the cross-link products and the IgG heavy chains (h.c.) are indicated. (**e**) Proteoliposomes reconstituted with purified Sec61 alone or together with purified endogenous SR and signal peptidase complex (SPC) were treated with BMH (10 μM) and analysed as in **a**.

To explore the interaction of SRP receptor with the translocon, we made use of a recombinant form of the SRP receptor, which lacks the N-terminal luminal and TM domains of SRβ (Fig. 2a), and which is functionally active in co-translational translocation^47^. In canine microsomes, SR is considerably sub-stoichiometric to Sec61 complex^45^, hence any SR-binding sites are unlikely to be saturated. Again, monitoring crosslinking from the single cysteine in Sec61β, we assessed the effect of adding recombinant SR on the environment of the Sec61 complex (Fig. 2b, lane 7). In the case of RMs, SRα/βΔN had little effect on the Sec61β crosslinking profile. In contrast, with EKRMs we observed a rather dramatic alteration in the cross-link profile on addition of SR. In particular, we observed a strong increase in a ∼80 kDa cross-link species, in good agreement with the expected size of an SRα × Sec61β adduct (Fig. 2b). This 80 kDa band was also observed with RM but to a much lesser extent. To assess whether the 80 kDa cross-link species observed in the presence of SRα/βΔN was indeed an SRα × Sec61β cross-link, we performed crosslinking and then reisolated recombinant SRα via the histidine (His)-tag using Ni-NTA resin, following membrane solubilisation (Fig. 2c). The 80 kDa cross-link species and the weaker band above this bound to the Ni-NTA resin, unlike Sec61β and all the other Sec61β cross-link products. The presence of the double band likely arises due to crosslinking of the single cysteine of Sec61β to one or the other of two different cysteines within SRα resulting in products with different electrophoretic mobility.

We also analysed cross-linked samples by western blot with Sec61β and SRα antiserum in parallel (Supplementary Fig. 1a). This clearly revealed that the strong 80 kDa species also cross-reacts with SRα antisera.

To test whether endogenous SR, that is, with the SRβ TM domain intact, could also be cross-linked to Sec61β, we treated EKRM with BMH and performed denaturing immunoprecipitation with anti-SRα antiserum (Fig. 2d). Again, an 80 kDa Sec61β cross-link species was detected and could be specifically immunoprecipitated with SRα antibodies but not with an unrelated antiserum.

Finally, we tested whether endogenous purified SR and Sec61 complex could also interact. Sec61 complex purified from pancreatic microsomes and reconstituted alone in liposomes and treated with BMH only revealed Sec61β cross-links to Sec61α, Sec61γ and homodimers of Sec61β (Fig. 2e). In contrast, proteoliposomes that also contained purified endogenous SR and signal peptidase complex (SPC) revealed an additional albeit weak 80 kDa Sec61β cross-link species in excellent agreement with the cross-link between SRα and Sec61 observed with recombinant SR and in EKRM.

### SRP receptor blocks Sec61β × Sec62 crosslinking

**Figure 3:**
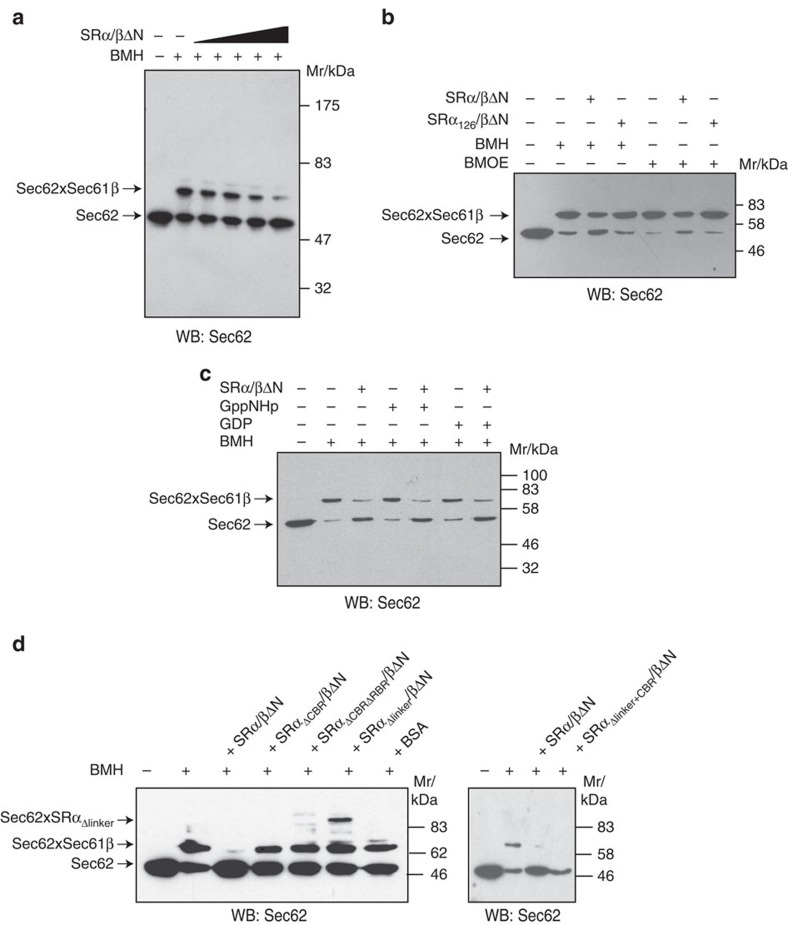
SR can displace Sec62 from Sec61β. (**a**) EKRM (32 eq) were preincubated with increasing concentrations of SRα/βΔN (750 nM, 1.5 μM, 3.75 μM and 7.5 μM), and then treated with BMH before analysis with SDS–PAGE and western blotting for Sec62. (**b**) EKRM were preincubated with either SRα/βΔN or SRα_126_/βΔN and then treated with BMH or bismaleimidoethane (BMOE) as indicated and analysed as in **a**. (**c**) EKRM were preincubated either alone or with SRα/βΔN in the absence of nucleotide or in the presence of either 10 mM GppNHp or 10 mM GDP, before crosslinking with BMH and analysis as in **a**. (**d**) EKRM were incubated with BSA, SRα/βΔN or SRα/βΔN harbouring the indicated linker mutations and then crosslinking was induced with BMH before analysis with SDS-PAGE and western blotting with anti-Sec62 antisera. The position of a novel cross-link observed between Sec62 and SRα_Δlinker_ is indicated.

In contrast to the Sec61β × SRα cross-link species, which increased in abundance when SR was added to EKRMs, most other Sec61β-derived cross-linked adducts were reduced, in particular, the cross-link between Sec61β and Sec62 (Fig. 2b). Moreover, this effect could be confirmed by pre-incubating EKRMs with increasing concentrations of SR, which revealed a dose-dependent inhibition in crosslinking between Sec62 and Sec61β (Fig. 3a). When this experiment was repeated using a mutant SRP receptor, which contains only the SRX domain of SRα (SRα_126_/βΔN, Fig. 2a)^5^, a much weaker reduction in Sec61β–Sec62 crosslinking was observed. This suggests that either the linker and/or NG regions are important for the inhibitory effect observed (Figs 2b and 3b). Similar results were obtained using the bifunctional reagent bismaleimidoethane, which has a shorter spacer arm between the reactive maleimides than BMH (10 and 16 Å, respectively; Fig. 3b).

As both SRα and SRβ are GTPases, we also assessed whether the inhibition of the Sec61–Sec62 interaction was dependent on guanine nucleotides. However, pre-incubation of SR and EKRMs with either GDP or the non-hydrolyzable analogue GppNHp (5′-guanylyl imidodiphosphate) had no effect on the SR-induced reduction of Sec61 × Sec62 crosslinking (Fig. 3c). Consistent with this result, crosslinking between HisSRα and Sec61β was also insensitive to guanine nucleotides (Supplementary Fig. 1b).

### SRα linker facilitates ribosome and translocon interaction

The fact that the inhibitory effect of SR was independent of nucleotides, led us to investigate whether the SRα linker domain, rather than the GTP-binding NG domain, might be responsible. Comparison of the linker region from different eukaryotes reveals two distinct charged regions, which we term CBR and RBR (Fig. 2a, Supplementary Fig. 2). A series of mutants were generated lacking either just the first one (ΔCBR), both charged domains (ΔCBRΔRBR) or the entire linker domain (Δlinker), but retaining the SRX and NG domains intact (Fig. 2a).

When the cross-link assay was repeated with these mutants, in contrast to the full-length SR, none of them could reduce the Sec61β × Sec62 product (Fig. 3d). These results strongly suggest that the linker domain is important for this effect. When the construct lacking the entire linker domain (residues 126–315) was used (SRα_Δlinker_), an additional cross-link adduct of 92 kDa was present corresponding precisely to the size of Sec62 plus SRα_Δlinker_.

To test whether the CBR region is sufficient for this effect, we performed the same experiment with a construct where the entire linker is replaced with just the CBR (Δlinker+CBR) (Fig. 3d). Indeed this construct was also able to reduce crosslinking between Sec61β and Sec62. Our data show that the first charged region is required for Sec62 displacement.

We also monitored membrane binding of the different SR linker mutants using EKRM in a floatation assay (Supplementary Fig. 3). Constructs with CBR (that is, full-length SR and Δlinker+CBR) showed the strongest membrane binding, consistent with the role of this domain in interacting with the translocon. Although the other mutants showed lower membrane binding, all of them could still interact with the membrane. In the case of the Δlinker construct, this is in good agreement with the observed cross-link between SRα_Δlinker_ and Sec62. This indicates that the inability of mutants lacking the CBR domain to perturb the Sec62–Sec61 interaction is not simply due to a loss of membrane or translocon interaction.

### SRα linker domain is important for ribosome binding of SR

**Figure 4:**
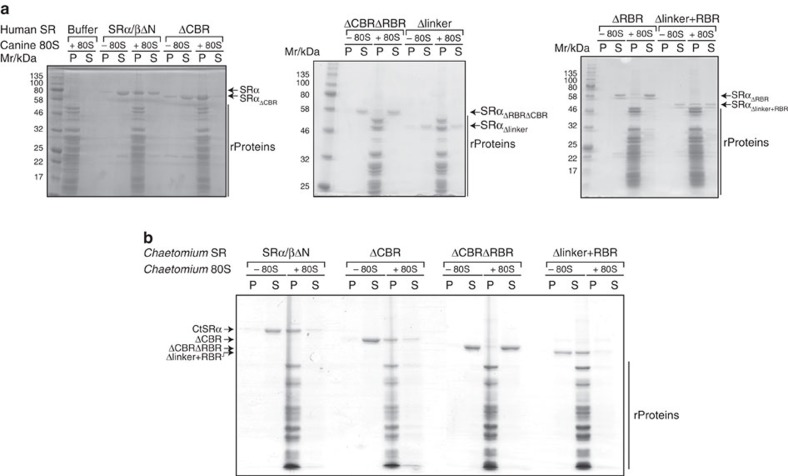
The conserved charged region of the SRα linker domain is involved in ribosome binding. (**a**) Indicated constructs of human SRα/βΔN were incubated in the presence and absence of salt-washed canine pancreatic ribosomes. Bound material was then recovered by sedimentation through a sucrose cushion and the supernatant (S) and pellet (P) fractions analysed by SDS–PAGE and staining with Coomassie Brilliant Blue. (**b**) Analysis as in **a** but with salt-washed ribosomes and SRα constructs from *Chaetomium thermophilum (Ct)*.

The linker domain is also known to be required for ribosome binding of mammalian SR^47^,^48^. We made use of the linker mutants to assess whether this property of SR might also depend on a specific region of the linker domain. Using a sedimentation assay with salt-washed canine pancreas ribosomes, binding of full-length SR to ribosomes could be observed as reported previously (Fig. 4a)^47^,^48^. Removal of both charged regions (ΔCBRΔRBR) or the entire linker (Δlinker) abolished ribosome binding; however, deletion of only the N-terminal region (ΔCBR) led to increased ribosome association. Deletion of just the RBR domain strongly reduced ribosome binding. In contrast, a construct where the entire linker is replaced with the RBR domain alone (Δlinker+RBR) still showed ribosome association, albeit not as efficient as full-length SR.

While the ability of SR to bind ribosomes appears linked to the RBR region of the linker domain, this contrasts to the effect of SR on Sec61β × Sec62 crosslinking, which is closely linked to the CBR region (Fig. 3d). Hence the linker domain appears to be involved in both binding the ribosome and destabilizing the Sec62–Sec61 interaction. However, these two functions require distinct regions of the linker domain.

### SR ribosome binding is evolutionarily conserved

Having observed that the linker domain harbours two distinct charged regions, we used multiple sequence alignments to further dissect these elements (Supplementary Fig. 2a). The CBR, which is involved in Sec62 displacement, is more specific to animals, while the RBR is evolutionarily well-conserved between fungi, plants and animals. As the latter was implicated in ribosome binding, the role of this region was further assessed using a fungal SR. Analogous linker domain mutations were generated in SRα from the thermophilic fungus *Chaetomium thermophilum* (Supplementary Fig. 2b) and their ability to bind to *Chaetomium* 80S ribosomes was tested (Fig. 4b). Compared with human SRα, full-length CtSRα is more stable in the absence of SRβ and allowed us to monitor ribosome binding independent of SRβ. Moreover, we recently showed that while CtSRα/SRβΔN can bind ribosomes, both CtSRβΔN alone and the minimal CtSRα_138_/SRβΔN complex are unable to bind ribosomes^26^. Full-length CtSRα alone readily bound to the ribosomes in a sedimentation assay (Fig. 4b), as well as to canine ribosomes (Supplementary Fig. 4b). Deletion of the non-conserved linker region (ΔCBR) had very little effect on ribosome binding. In contrast, deletion of the conserved RBR region as well almost completely abolished ribosome binding (ΔCBRΔRBR). Replacing the entire linker with just the RBR domain completely restored ribosome binding (Δlinker+RBR). Hence the role of the RBR is conserved between yeast and mammals.

Unlike the ribosome-binding activity of SRα, CtSRα was unable to destabilize the interaction between Sec61β and Sec62 (Supplementary Fig. 4c), suggesting that this interaction is specific to higher eukaryotes. Interestingly, the CtSRα NG domain alone could also be cross-linked to Sec62, as was observed with the human SRα_Δlinker_/βΔN construct (Supplementary Fig. 4c), indicating that the NG–translocon interaction is conserved.

### SR inhibits translocation of Sec62-dependent substrates

**Figure 5:**
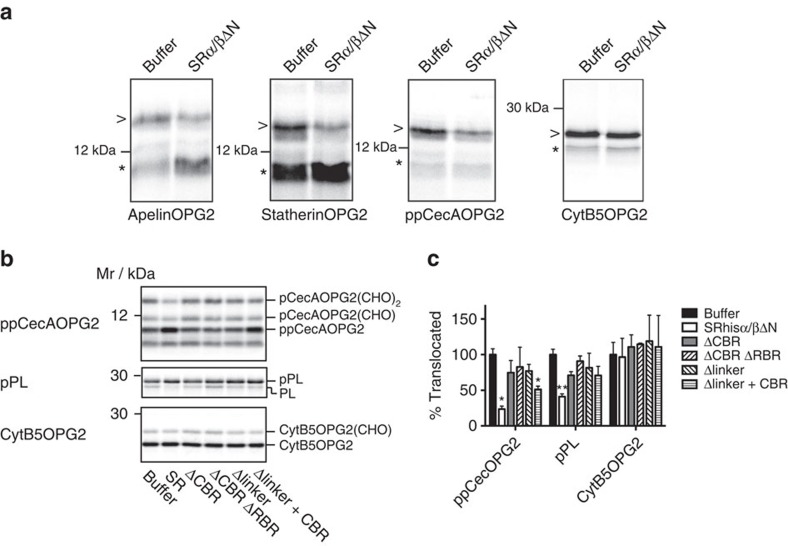
SR can inhibit translocation of Sec62-dependent precursors. (**a**) Constructs of apelin, statherin, preprocecropin A (ppcec A) and cytochrome B5 (cyt B5) each with a C-terminal opsin tag containing two N-linked glycosylation sites (OPG2) were translated *in vitro* in rabbit reticulocyte lysate in the presence of [^35^S] methionine. Synthesis was terminated with puromycin to ensure release of all nascent chains from the ribosome. PKRM were then added in the presence of absence of SRα/βΔN (10 μM) and then incubated at 30 °C to permit targeting and translocation. Membranes were then reisolated through a sucrose cushion and analysed by SDS–PAGE and phosphorimaging. The position of unglycosylated non signal-sequence cleaved (*) and signal-sequence cleaved, twice glycosylated species (>) is indicated. (**b**) Preprocecropin A (ppCec A) and cytochrome B5 both with a C-terminal opsin tag (OPG2) as well as preprolactin (pPL) were translated in reticulocyte lysate in the presense [^35^S] methionine and microsomes that had been preincubated with either buffer or the different SR constructs. Processed and non-processed forms of each precursor were recovered by denaturing immuno-precipitation and analysed by SDS–PAGE and phosphorimaging. (**c**) Relative translocation efficiency was determined from the ratio of processed to non-processed form for each precursor (as in **b**). Translocation in the absence of recombinant SR was set to 100%. Data are the means of three independent experiments. Error bars represent s.e.m. Differences significant from the buffer control are indicated (one-way analysis of variance, *P<0.05, **P<0.01).

Having observed that human SR can perturb the association between mammalian Sec62 and Sec61, the functional effect of SR on the translocation of Sec62-dependent substrates was tested. Insect preprocecropin A has long been known to translocate in an SRP-independent manner and was recently shown to instead require Sec62 (refs 37, 49). Similarly, the small mammalian secretory proteins apelin and statherin also translocate in a Sec62-dependent manner^49^. Apelin, statherin and preprocecropin A derivatives, possessing a N-glycosylation opsin tag to monitor ER translocation, were synthesised *in vitro* in rabbit reticulocyte lysate in the presence of labelled methionine. To exclude the possibility of any co-translational translocation, the translation reaction was treated with puromycin before the addition of RMs in the presence or absence of SRα/βΔN. Precursor translocation was then monitored via N-linked glycosylation. All three substrates showed a marked decrease in translocation in the presence of SRα/βΔN (Fig. 5a). In contrast, the C terminus of the tail-anchored membrane protein cytochrome B5–opsin construct, which can insert into the ER membrane spontaneously^50^, was N-glycosylated equally efficiently in the presence or the absence of SR, indicating that the loss of N-linked glycosylation of the short secretory proteins is not due to an indirect effect of SR on oligosaccharyltransferase activity.

To further investigate the specificity of this perturbation, we compared the inhibitory effect of the different SR linker deletion constructs on preprocecropin translocation. We performed the analysis in a co-translational reaction so that we could monitor effects on the SRP-dependent translocation of preprolactin in parallel (Fig. 5b,c). Control reactions performed in the absence of membranes or in the presence of membranes followed by treatment with Endo H confirmed the identity of the translocated species (Supplementary Fig. 5).

As with the post-translational assay, full-length SR led to a significant inhibition of preprocecropin translocation. In contrast, the ΔCBR, ΔCBRΔRBR and Δlinker constructs all failed to block translocation. The construct where the linker is replaced by the CBR domain alone (Δlinker+CBR) also inhibited translocation, although not quite as strongly as full-length SR. These results are, therefore, in close agreement with the ability of the different linker mutants to effect the Sec61–Sec62 interaction.

Full-length SR was also able to reduce translocation of preprolactin, although the effect was much weaker than observed for preprocecropin. This is consistent with previous studies using endogenous SR and Sec61 complex reconstituted into proteoliposomes, which also showed that increasing levels of SR relative to Sec61 reduced translocation of the efficiency of preprolactin^45^.

Importantly, none of the linker mutants were able to inhibit preprolactin translocation. The lack of effect of the Δlinker+CBR construct, which did block preprocecropin translocation, therefore indicates distinct underlying mechanisms of inhibition for the two pathways. Neither the full-length SR nor any of the linker mutants had any effect on cytochrome B5 integration.

## Discussion

Here we have shown that the interaction of Sec62 and Sec61 as revealed by crosslinking is highly sensitive to the presence and the absence of ribosomes. In contrast to the core Sec61 heterotrimer, the heptameric yeast Sec complex has been shown to be unable to bind to ribosomes^51^. Consistent with this, low resolution cryo-electron microscopy structures of the Sec complex reveal additional density above the cytoplasmic face of the core Sec61 heterotrimer^52^, which would likely occupy a similar position as the ribosome^53^ and hence occlude binding. This is in good agreement with the observation that crosslinking between mammalian Sec62 and Sec61β is strongly induced by the removal of ribosomes from RMs and is inhibited by their readdition. Furthermore, cross-links between Sec62 and Sec61β were shown previously to be exclusively absent from the ribosome-associated membrane protein fraction following detergent solubilisation^34^,^35^.

The fact that more than 50% of Sec62 can be cross-linked to Sec61, following removal of ribosomes, suggests that the interaction of these components is strongly favoured if ribosomes are absent. Furthermore, if the free Sec complex is unable to interact with ribosomes^51^, this raises the question as to how ribosomes can subsequently rebind to such complexes. Here we find that SR is able to destabilize the interaction between Sec62 and Sec61, which we detect as a loss of crosslinking between Sec62 and Sec61β and a block in the translocation of Sec62-dependent substrates.

**Figure 6:**
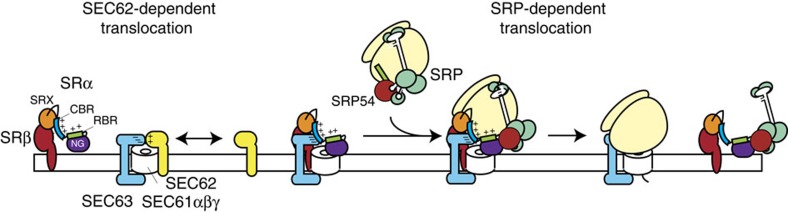
Model depicting distinct roles of SRα linker domain in Sec62 displacement from Sec61 and ribosome recruitment. During Sec62-dependent translocation cytosolic chaperones maintain the precursor in an unfolded translocation-competent state (upper panel). Precursors are delivered to the Sec translocon comprising Sec61 complex associated with Sec62 and Sec63. The latter are thought to interact with one another electrostatically via charged domain within the two proteins. Translocation is driven by ATP hydrolysis of the luminal Hsp70 chaperones. SRP–RNC complexes are unable to interact with the larger Sec translocon. During SRP-dependent translocation, SR can interact with the Sec translocase displacing Sec62 (lower panel). This requires the CBR of the SRα linker domain, which may compete with Sec62 for binding to the charged regions of Sec63 and/or Sec61. This displacement of Sec62 allows approach by RNC–SRP complexes, which can bind the SRP receptor via interactions of the NG domains of SRP54 and SRα and components of the ribosome with the charged RBR domain of the SRα linker region. Hence SR can preferentially recruit SRP–RNC complexes and then transfer them to the Sec61 translocon.

It is well established that SR catalyses the transfer of incoming ribosome–nascent chain complexes from SRP to the Sec61 translocon^11^. Hence, the ability of SR to interact with Sec61 and displace or reposition Sec62, immediately suggests a mechanism by which the Sec complex can be reorganized to permit the subsequent docking of the ribosome–nascent chain complex (Fig. 6). Furthermore, the SR–Sec61 intermediate would preferentially recruit SRP-bound ribosome–nascent chain complexes, potentially providing an additional step where targeting fidelity can be enhanced. Moreover, this would also rationalize the previous observation that SRP-bound ribosome–nascent chains can access a pool of Sec61 to which free ribosomes are unable to bind^54^.

A recent study showed that arresting translocation of a co-translational substrate using a small, tightly folded domain at the luminal side of the translocon, led to the stable recruitment of Sec62 (ref. 55). Hence, although dispensable for co-translational translocation of most substrates^37^,^38^,^39^, a subset of nascent chains may require Sec62 to facilitate later steps of their the translocation. Such nascent chains may possess particular features, which initiate reorganization of the ribosome–translocon complex and thereby expose a stable binding site for Sec62.

Our experiments revealed that crosslinking of SRα to Sec61β was much stronger with PKRMs as compared with RMs. There is still a low amount of SRα to Sec61β crosslinking in RM, rationalised by the fact that ∼80–90% of translocons are engaged with ribosomes^45^, hence a small population of translocons are still available to bind SR.

SR also had little effect on the overall cross-link profile of Sec61β with RMs as compared with PKRMs. This suggests that once the ribosome is fully engaged with the translocon, access of SR is likely to be strongly hindered. This is rationalized by cryo-electron microscopy structures of the ribosome–Sec61 (ref. 53) and ribosome–SRP–SR^6^ complexes, which predict a strong potential overlap of density at the ribosomal exit tunnel.

Complete deletion of the linker domain led to the formation of a novel cross-link between SRα and Sec62. This is also observed with the CtSRαNG domain, indicating that this interaction from the NG domain appears conserved. Human and *Chaetomium* NG domains both contain multiple cysteines but only one of these is conserved and is located in helix 3 of the N-domain, hence this is a strong candidate to be involved in crosslinking to Sec62. *Escherichia coli* FtsY, which lacks the linker domain, is known to interact with cytoplasmic loop 5 of SecY via its NG domain. Therefore, in the absence of the linker, SRα may also bind to the Sec61 complex in a similar manner, positioning the NG domain close to Sec62. If the linker is still present, but unable to displace Sec62, as with full-length CtSRα, this may interfere with the binding of NG domain.

As well as interacting with the translocon, SRα also binds to the ribosome^47^,^48^. Our study characterizes a long stretch of positively charged residues in the linker region of eukaryotic SRα of previously unknown function. We identify two distinct elements within the linker and show that ribosome binding requires the second element (RBR) that is conserved between fungi and mammals. Notably, a large number of factors that act on the nascent polypeptide use the tunnel exit as a hub for interaction. Although these factors are unrelated in structural and functional terms, they seem to use a common mechanism for ribosome interaction involving a stretch of positively charged residues^56^,^57^. The RBR identified in this study within the linker region of SRα probably acts by the same mechanism.

The first element (CBR) being unique to higher eukaryotes and rich in lysine residues is necessary and sufficient for the displacement of Sec62 by SRα. Sec62 contains several positively charged regions also rich in lysine residues. Hence the linker of SRα and Sec62 are likely to compete for Sec translocon binding via electrostatic interactions. Indeed, Sec63 has a very negatively charged C terminus^34^,^35^ and so might well contribute to the mutually exclusive binding site. In this respect, CBR and Sec62 might also compete for binding to Sec63.

Interestingly, mammalian Sec62 differs from its yeast homologues by the presence of an additional positively charged region in the N-terminal region^58^ (Supplementary Fig. 6). This might represent an extra level of regulation in higher eukaryotes, and explain why the additional positive-charged (CBR) region within the linker domain of human SR is required to displace Sec62 from Sec61.

Taken together, we have identified two distinct interaction motifs in SR and show that one of them serves a function that is distinct from the ‘canonical' eukaryotic RBR in regulating association of Sec62 with Sec61 in the ER membrane. Cells under different physiological conditions can express different levels of SR^59^. In combination with our findings, this may provide a mechanism to differentially modulate the flux of substrates through two distinct ER translocation pathways, allowing cells to efficiently respond to changing profiles of secretory substrates with distinct properties under different physiological conditions.

## Methods

### Purification of mammalian SR and deletion variants

For purification of mammalian SR, a bi-cistronic construct containing human SRα and murine SRβΔTM was cloned in the pET16b vector (Novagen). Linker deletion variants of SR were created using the same construct. Expression was performed in *E. coli* BL21 (DE3) cells with overnight induction with 1 mM isopropyl-β-D-thiogalactoside at 16 °C. The cells were lysed using a sonicator followed by micro fluidizer in SR buffer (25 mM HEPES pH (7.5), 300 mM KOAc, and 5 mM Mg(OAc)_2_) and cleared by centrifugation at 63,000 g for 30 min using a JA25.50 (Beckman) rotor. The resulting supernatant was purified first by Ni-NTA affinity chromatography. The eluate was applied to a Q-sepharose column and the flow-through, then bound to SP-sepharose resin and eluted with a 0.2–1 M KOAc salt gradient in SR buffer. Finally, the SR was purified by size exclusion chromatography on a Superdex 200 column^47^. The final concentration of salt was reduced to 150 mM before snap freezing in liquid nitrogen.

### Purification of CtSR and linker deletion variants

CtSRα and the deletion variants and CtSRβ were cloned as hexa-histidine-tagged proteins via the pETHis vector^26^,^60^. Untagged CtSRβΔTM was cloned into the pET24a vector (Novagen). CtSR His-tagged CtSRα and untagged CtSRβΔTM were expressed individually using the auto-induction method in *E. coli* BL21 (DE3) cells at 24 °C overnight^61^. Pellets of cells expressing CtSRα and CtSRβΔTM were lysed together in the lysis buffer (20 mM Tris pH 8.0, 150 mM NaCl, 5 mM MgCl_2_, 20 mM imidazole and 0.02% (v/v) Nonidet-P40 ) using a microfluidizer. After the lysis, the cell debris was removed by centrifugation at 67,000 *g* using JA25.50 (Beckman) rotor. The supernatant was collected and incubated at 4 °C for 1 h on a rotating wheel. The CtSR complex was then purified as described for mammalian SR^47^. His-tagged CtSRα, CtSRα deletion variants and CtSRβΔTM were expressed and purified similarly.

### Microsome crosslinking in the presence of SR

RMs, EKRMs and PKRMs were prepared from canine pancreas^62^,^63^. Pancreatic tissue was cut into small pieces, passed through a tissue press and then homogenized in RM buffer (20 mM HEPES–KOH pH 7.6, 50 mM KOAc, 2 mM Mg(OAc)_2_, 250 mM sucrose and 2 mM DTT). The resulting extract was then centrifuged sequentially at 1,000 *g* and 10,000 *g* to yield a post-mitochondrial supernatant, which was overlayed onto step gradients of 1.5, 1.75 and 2 M sucrose (in RM buffer) and centrifuged overnight at 235,000 *g* at 4 °C using a Ti45 rotor (Beckman). Membranes banding at the 1.75–2 M cushion interface were collected, diluted with RM buffer without sucrose (RM−) and pelleted before resuspension in RM buffer at a concentration of 4 eq μl^−1^ to yield RMs.

EKRMs were prepared by washing RMs with high salt (650 mM KOAc) before resuspension in 20 mM HEPES–KOH pH 7.6, 20 mM EDTA, 650 mM KOAc, 2 mM DTT and 2 M sucrose. Membranes were held on ice for 30 min and then overlayed with sucrose cushions of 1.5, 1 and 0.25 M in the same buffer and centrifuged overnight in the SW40 rotor (Beckman) at 38,000 r.p.m. Membranes floating at the 0.25–1 M interface were collected, diluted with RM(−) buffer and collected by centrifugation before resuspending in RM buffer to give EKRM. PKRM were prepared in an identical manner, except salt-washed membranes were resuspended in 20 mM HEPES–KOH pH 7.6, 500 mM KOAc, 5 mM Mg(OAc)_2_, 2 mM puromycin, 2 mM GTP and 2 M sucrose (prewarmed to 25 °C), and incubated at 25 °C for 20 min before floatation.

To purify translocon components, RMs were pre-extracted with 0.1% (w/v) digitonin and then solubilised with 3% (w/v) digitonin in the presence of 500 mM KOAc. The resulting extract was then centrifuged to yield a ribosome pellet and supernatant. To purify SPC, the supernatant was applied to a concanavilin A column. Bound proteins were eluted with α-methylmannoside and then SPC purified on an SP-sepharose ion exchange column^45^. SR was purified from the flow-through of the concanavalin A column using an anti-SRα immunoaffinity column^45^,^64^. Sec61 was purified from the ribosome pellet fraction by ion-exchange chromatography with Q and SP-sepharose, following release from the ribosome by puromycin treatment^45^,^64^.

Purified components were detergent exchanged from digitonin into 0.2% (w/v) deoxyBigCHAP on a SP-sepharose column and reconstituted into liposomes by combining with 10 mg ml^−1^ phosphatidlycholine:phosphatidlyethanolamine (4:1) and incubated overnight with SM2 Biobeads (Bio-Rad)^45^,^64^.

Ten equivalents of EKRMs or PKRMs were incubated with 0.7–10 μM of mammalian SR and linker deletion variants in a 20 μl reaction in the assay buffer (25 mM HEPES–KOH pH 7.5, 120 mM KOAc, 2 mM Mg(OAc)_2_, 250 mM sucrose) at 25 °C for 10–15 min. BMH was added to the final concentration of 10 μM and the reaction mixture was further incubated at 25 °C for 10 min. The crosslinking reaction was stopped by the addition of 1 mM DTT and incubating the sample on ice for 15 min. The whole sample was then mixed with SDS sample buffer and proteins and crosslinking adducts were analysed by SDS–PAGE and western blotting with either Sec61β (1:3,000), SRα (1:1,000) or Sec62 (1:1,000) antibodies. Complete blot images for cropped panels are shown in Supplementary Fig. 7.

For immunoprecipitation of endogenous cross-linked SRα, EKRM (100 eq) were treated with 40 μM BMH for 10 min at 30 °C. After quenching with 10 mM DTT, membranes were reisolated by centrifugation and then resuspended in 70 μl of 20 mM Tris-HCl pH 8.0. 2% (w/v) SDS and 2 mM EDTA and heated for 10 min at 70 °C. The denatured membranes were diluted with 700 μl of ice-cold immunoprecipitation (IP) buffer A (20 mM Tris-HCl pH 8.0, 0.4% (v/v) Nonidet-P40, 150 mM NaCl, 1 mM EDTA, 1 mM PMSF (phenylmethylsulfonyl fluoride)) and then 2 μl of anti-SRα or a non-related rabbit antiserum were added. After rolling for 1 h at 4 °C, immune complexes were recovered with protein A-sepharose (Genscript) and then washed two times with IP buffer A, two times with 20 mM Tris-HCl pH 8.0, 0.2% (v/v) Triton X-100, 500 mM NaCl, 1 mM EDTA and once with 10 mM Tris-HCl pH 8.0, before elution with SDS–PAGE sample buffer at 70 °C for 10 min. Samples were then analysed by SDS–PAGE and western blot with Sec61β antibodies (1:1,000) and detection with protein A–horseradish peroxidase (Sigma) to reduce cross-reaction with IgG heavy chains.

### Preparation of Canine and *Chaetomium* 80S ribosomes

To prepare canine ribosomes^47^, dog RM were resuspended in 50 mM HEPES–KOH pH 7.6, 600 mM KOAc, 12 mM Mg(OAc)_2_, 2 mM DTT, with protease inhibitor cocktail and treated with 0.5 mM puromycin for 15 min at 25 °C. The reaction was then overlayed onto a 1.5 M sucrose cushion made up in the same buffer and centrifuged at 444,000 *g* for 1 h 20 min at 15 °C in the MLA-80 rotor (Beckmann). The resulting ribosomal pellet was then rinsed and resuspended in 25 mM HEPES–KOH pH 7.6, 120 mM KOAc, 2 mM Mg(OAc)_2_, 1 mM DTT.

For purification of *Chaetomium* ribosomes^56^, *C. thermophilum* cells were grown in a rotary shaker at 90 r.p.m. at 55 °C for 3 days. Cells were then harvested with a vacuum filter and ground to a fine powder in a mortar in the presence of liquid nitrogen. The powdered mycelium was then resuspended in 20 mM HEPES–KOH (pH 7.5), 100 mM potassium acetate, 125 mM sucrose, 7.5 mM Mg(OAc)_2_, 1 mM DTT and 0.5 mM PMSF and centrifuged at 28,000 *g* in SS-34 rotor (Sorvall), for 15 min to remove the insoluble material. To pellet ribosomes, the supernatant was overlaid on a high-salt sucrose cushion (500 mM potassium acetate, 1.5 M sucrose) prepared in the same lysis buffer and centrifuged at 300,000 *g* in a Ti70 rotor (Beckman) for 18 h. The translucent ribosomal pellet of ribosomes was then resuspended in 20 mM HEPES–KOH (pH 7.5), 120 mM potassium acetate, 5 mM magnesium acetate, 1 mM DTT and 0.5 mM PMSF.

### Ribosome co-sedimentation assay

For the co-sedimentation assay, 25 pmol of ribosomes was incubated with 100 pmol of each protein in the assay buffer containing (25 mM HEPES pH 7.5, 250 mM KOAc, 2 mM Mg(OAc)_2_, 0.05% (v/v) Triton X-100 and 1 mM DTT). The reaction mixture was incubated at 25 °C for 20 min and then loaded onto a 0.5 M sucrose cushion prepared in the assay buffer. Ribosomes were pelleted by ultracentrifugation at 267,000 *g* at 4 °C for 1 h using a TLA100.3 rotor (Beckman). The proteins in the pellet fraction and in the supernatant (following precipitation with trichloroacetic acid) were analysed using SDS–PAGE and staining with Coomassie Brilliant Blue.

### *In vitro* translocation assays

Templates for preprolactin^65^, opsin-tagged preprocecropin A, apelin, statherin^39^ and cytochrome B5^66^ were generated by PCR^39^. Transcription reactions were carried out in a volume of 100 μl for 2 h at 37 °C, in the presence of 1 × transcription-optimised buffer (Promega), 10 mM DTT, 0.5 μg of template DNA, 0.25 mM each of rATP, rCTP, rGTP and rUTP (Promega), 0.5 mM Cap analogue (m^7^G[5′]ppp[5′]G; New England Biolabs), 80 units of T7 RNA Polymerase (Promega) and 100 units of RNasin Plus RNase Inhibitor (Promega). Transcripts were then purified with an RNeasy Mini kit (Qiagen) according to the manufacturer's instructions.

For post-translational translocation assays, translation reactions (25 μl) were carried out using nuclease-treated rabbit reticulocyte lysate (Promega). Translations were performed in the presence of [^35^S] methionine (0.769 MBq, 43.48 TBq mmol^−1^; Perkin Elmer). Amino acids minus methionine (Promega) were added to 30 μM. 1 μg of *in vitro*-transcribed RNA was then added and the sample was incubated for 15 min at 30 °C. Puromycin was added to 1 mM following the translation and incubated at 30 °C for a further 5 min to ensure effective release of the polypeptide from the ribosome. Recombinant SR was added to 10 μM as well as 10 eq of RM and the sample was then incubated for 20 min at 30 °C. Membranes were recovered by centrifugation through an 80 μl high-salt cushion (0.75 M sucrose, 0.5 M KOAc, 5 mM Mg(OAc)_2_, 50 mM HEPES–KOH, pH 7.9) at 100,000 *g* for 10 min at 4 °C in the TLA100 rotor (Beckman). The membrane pellet was resuspended in 20 μl low-salt buffer, 100 mM sucrose, 100 mM KOAc, 5 mM Mg(OAc)_2_, 50 mM HEPES–KOH pH 7.9, 1 mM DTT and treated with 250 μg ml^−1^ RNase A at 37 °C for 10 mins. The resulting samples were analysed by SDS–PAGE and phosphorimaging using a Typhoon FLA-7000 (GE Healthcare).

For co-translational reactions, RMs were preincubated with 10 μM of the indicated recombinant SR (or an equal volume of SR buffer) on ice for 20 min and were then recovered by spinning at 100,000 *g* for 20 min and resuspended in RM buffer (250 mM sucrose, 50 mM HEPES–KOH pH 7.5, 50 mM KOAc, 2 mM Mg(OAc)_2_, 1 mM DTT). Translation reactions (25 μl) were carried out as above in the presence of 10 eq of SR/buffer-treated RMs and the samples were incubated for 30 min at 30 °C. Translated products were then immunoprecipitated by adding 10 volumes of Triton IP buffer (10 mM Tris-HCl pH 7.5, 140 mM NaCl, 1 mM EDTA, 1% (v/v) Triton X-100) and the appropriate antisera (anti-opsin^67^ or anti-pPL (a gift from Sharon Tooze)). The resulting samples were analysed by SDS–PAGE and phosphorimaging using a Typhoon FLA-7000 (GE Healthcare). Data were quantified using Aida (Raytek) and statistical analysis (one-way analysis of variance with Dunnett's *post hoc* test) was performed using GraphPad (Prism).

### Antibodies

The following antibodies, raised in rabbits against the indicated epitopes, were used: Sec61β (PGPTPSGTNC)^45^, SRβ (CADIQDLEKWLAKIA)^64^,SRα (KKFEDSEKAKKPVRC)^45^, Sec62 (a gift from R. Zimmermann—DGETPKSSHEKS)^36^ and Sec62 (Sigma *HPA014059*—residues 257–395), as well as a mouse monoclonal anti-opsin tag (GPNFYVPFS) antibody^67^. The uncropped immunoblots are provided in Supplementary Fig. 7.

## Additional information

**How to cite this article:** Jadhav, B. *et al*. Mammalian SRP receptor switches the Sec61 translocase from Sec62 to SRP-dependent translocation. *Nat. Commun.* 6:10133 doi: 10.1038/ncomms10133 (2015).

## Supplementary Material

Supplementary Information Supplementary Figures 1-7 and Supplementary Reference
