# Supplementary material for: Mammalian SRP receptor switches the Sec61 translocase from Sec62 to SRP-dependent translocation
Source: Nat Commun. 2015 Dec 4;6:10133. doi: 10.1038/ncomms10133 (PMC4686813; doi:10.1038/ncomms10133)
Supplement: Supplementary Information — Supplementary Figures 1-7 and Supplementary Reference [file ncomms10133-s1.pdf]

Supplementary Figures

a

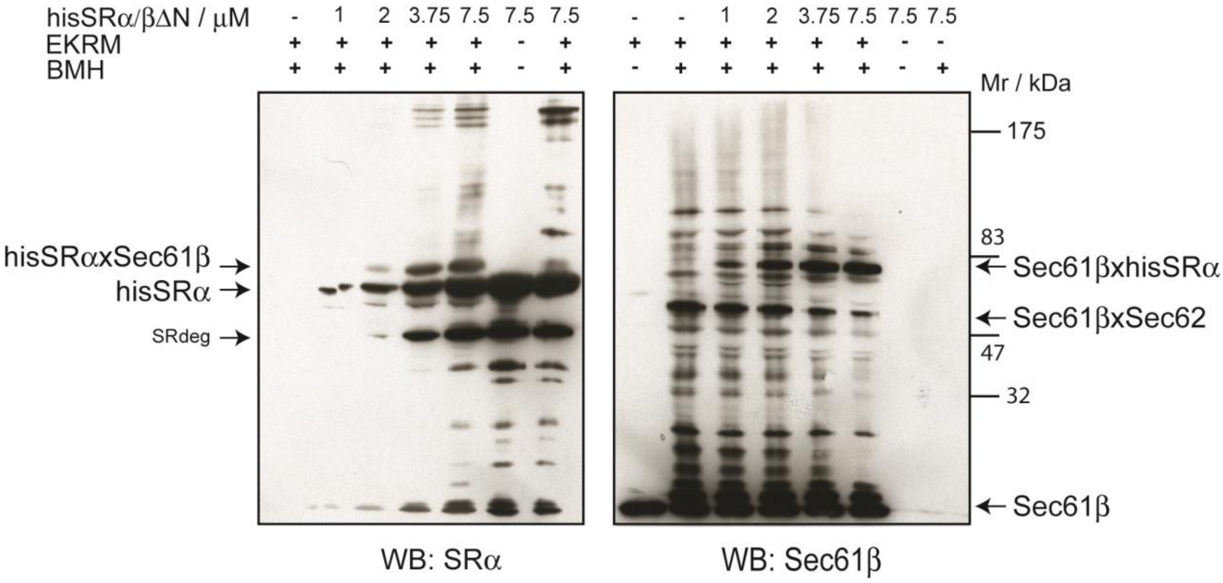

b

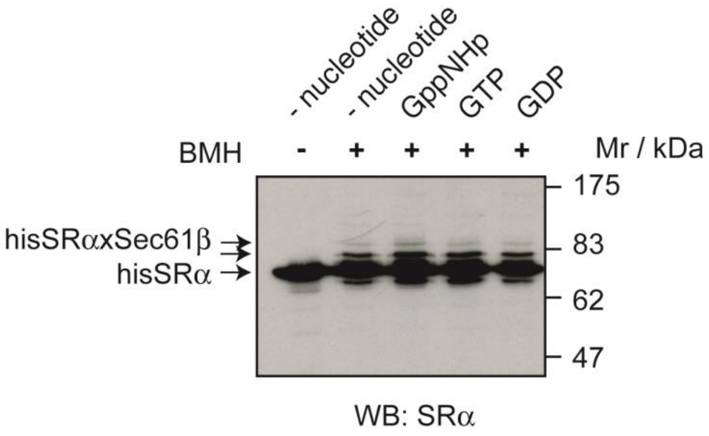

Supplementary Figure 1 SRα binds the Sec61 translocase in proximity to Sec61β

a. EKRM (32 eq) or buffer were incubated with the indicated concentrations of hisSRα/βΔN. Cross-linking was then induced with BMH (10 μM) and samples analysed in parallel on the same SDS-PAGE gel followed by Western blot with SRα or Sec61β antisera. A degradation product of SRα, which contains the SRα antibody epitope is indicated (SR-deg).

b. hisSRα/βΔN (2 μM) was incubated with EKRM in the presence of the indicated nucleotides (1 mM). Cross-linking was then induced with BMH (10 μM) and samples were then analysed by SDS-PAGE gel followed by Western blot with SRα antisera.

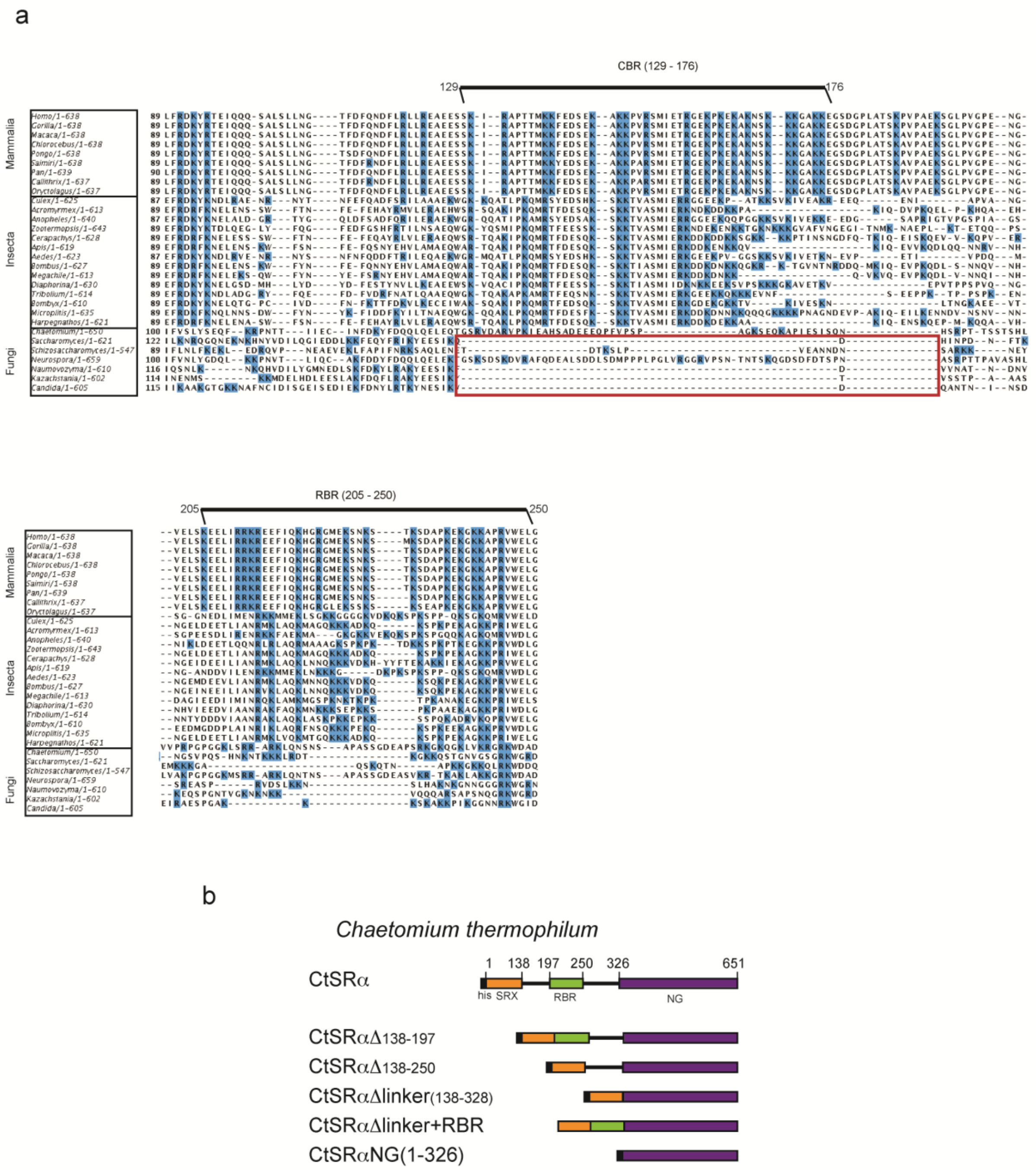

Supplementary Figure 2 Alignment of SRα linker domains

a. Linker domain regions of SRα from indicated eukaryotic species was compared using Clustal omega [1]. Positions of the mammalian-specific CBR and conserved RBR sub-domains are indicated by a bar on the top (numbering refers to human SRα).

b. Schematic of linker domain mutant constructs for *Chaetomium* SRα. Note that the RBR domain is conserved between Human and *Chaetomium* whereas the CBR domain is not.

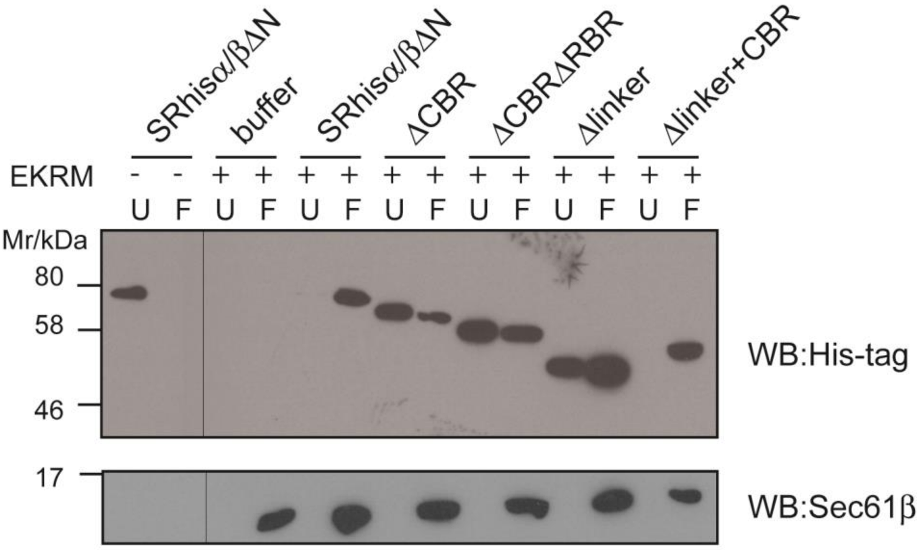

Supplementary Figure 3 SRα Linker domain mutants still associate with membranes

hisSRα/βΔN and the indicated linker domain mutants were incubated either with buffer or EKRM for 15 minutes at 25ºC. Membranes were then recovered by flotation through a Nycodenz cushion. Floated (F) and unfloated (U) fractions were analysed by SDS-PAGE and western blot using anti-his-tag and anti-Sec61β antibodies.

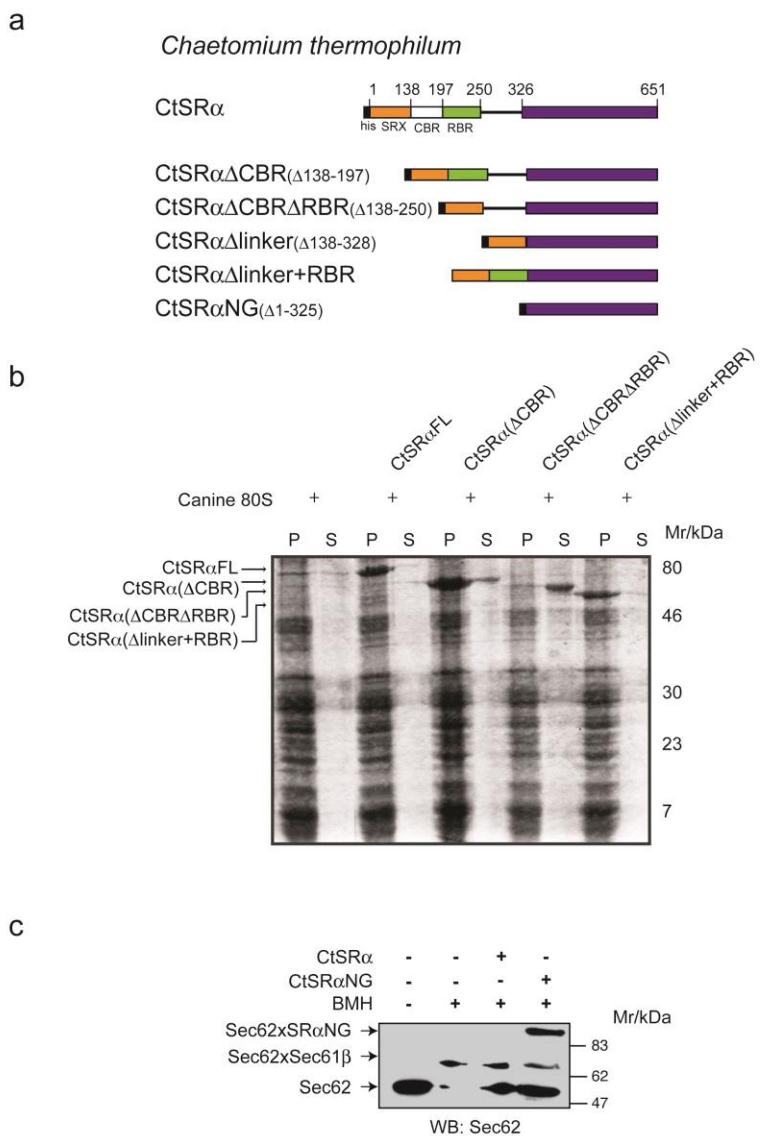

Supplementary Figure 4 Interaction of *Chaetomium thermophilum* (Ct) SRα with mammalian ribosomes and Sec62.

a. Schematic of *Chaetomium thermophilum* (Ct) SR truncation constructs.

b. Full length CtSRα and indicated linker mutants were purified and then incubated with high-salt washed canine pancreatic ribosomes. Following binding, ribosomes were recovered by sedimentation and then ribosome-enriched pellet (P) and supernatant (S) fractions were analysed by SDS-PAGE and staining with Coomassie-Brilliant Blue.

c. Full length CtSRα and CtSRαNG were purified and then incubated with EKRM prior to cross-linking with BMH. Samples were analysed by SDS-PAGE and western blot with anti-Sec62 antiserum.

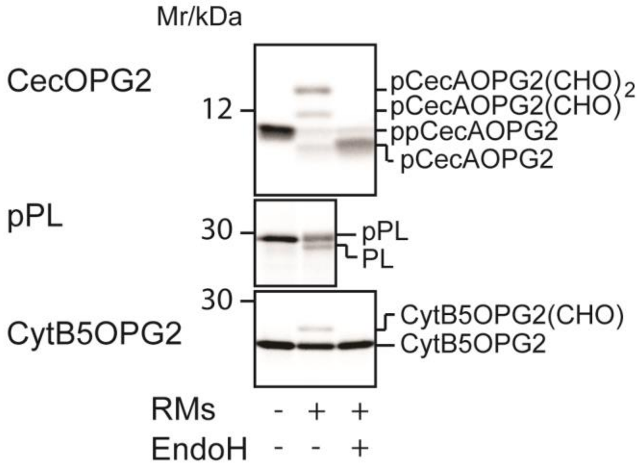

Supplementary Figure 5 Identity of translocated precursors.

PreprocecropinA-OPG2 (CecOPG2), cytochrome B5-OPG2 (CytB5OPG2) and preprolactin (pPL) were translated *in vitro* in the presense or absence of microsomes (RM) and then where indicated treated with Endo H to remove any N-linked glycans. Samples were recovered by immunoprecipitation using anti-opsin tag or anti-pPL antibodies and analysed by SDS-PAGE and phosphorimaging.

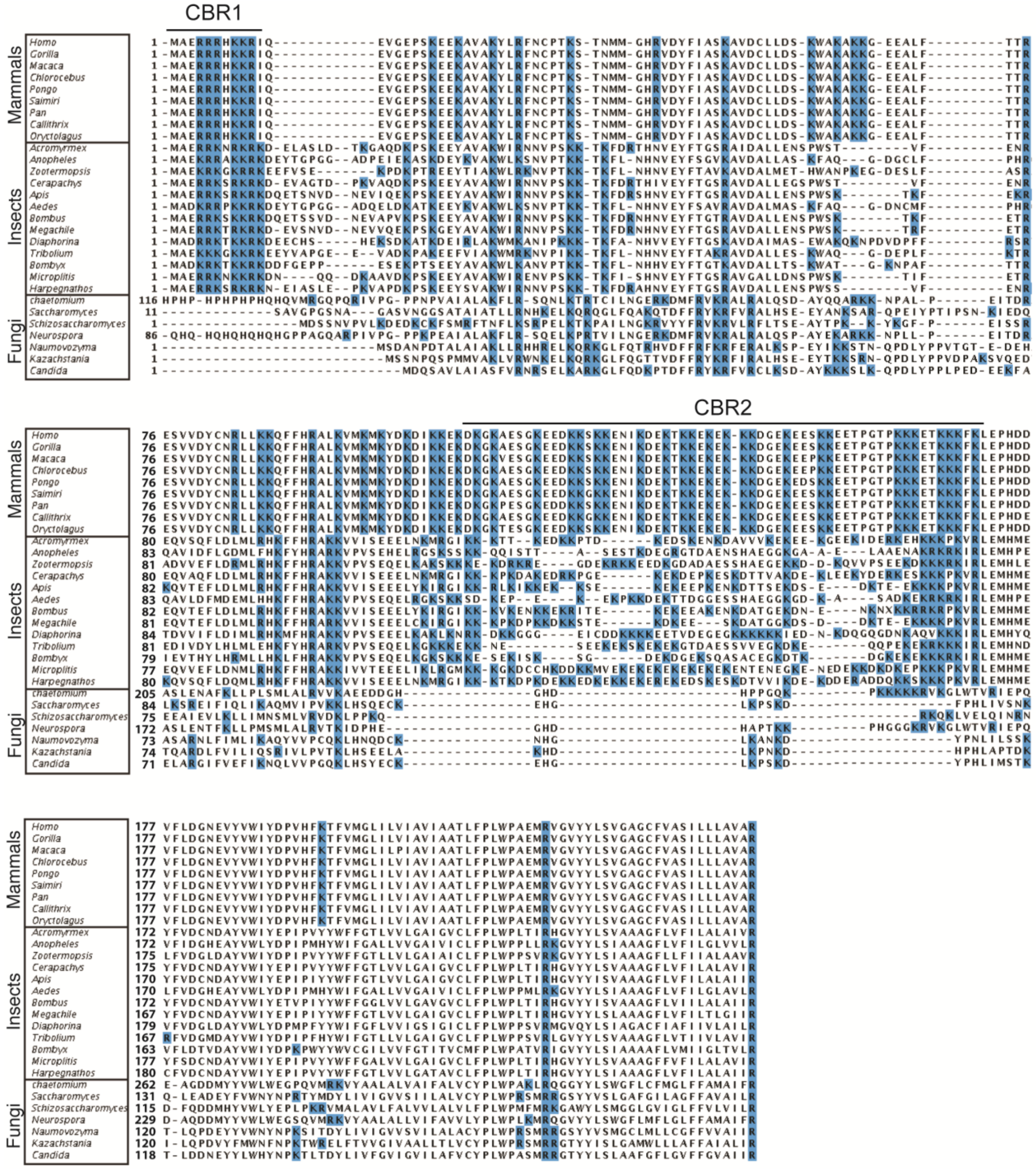

Supplementary Figure 6 Alignment of Sec62 charged domains

Positively charged regions of Sec62 from indicated eukaryotic species were compared using Clustal omega [1]. Positions of the metazoan-specific CBR1 and CBR2 domains are indicated by bars.

Figure 3c complete blot image

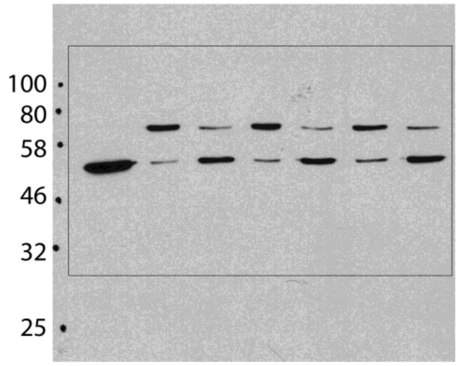

Figure 3d complete blot images

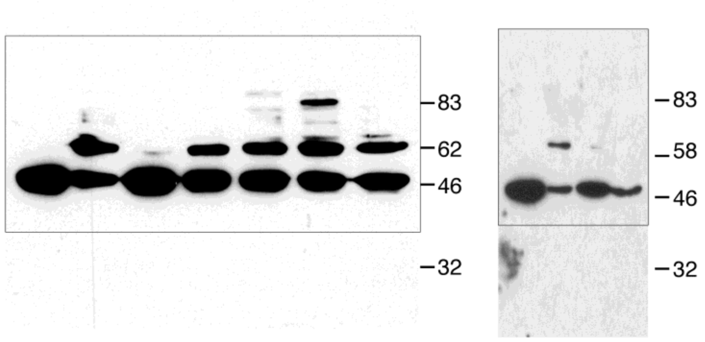

Figure 5a complete phosphorimager gel images

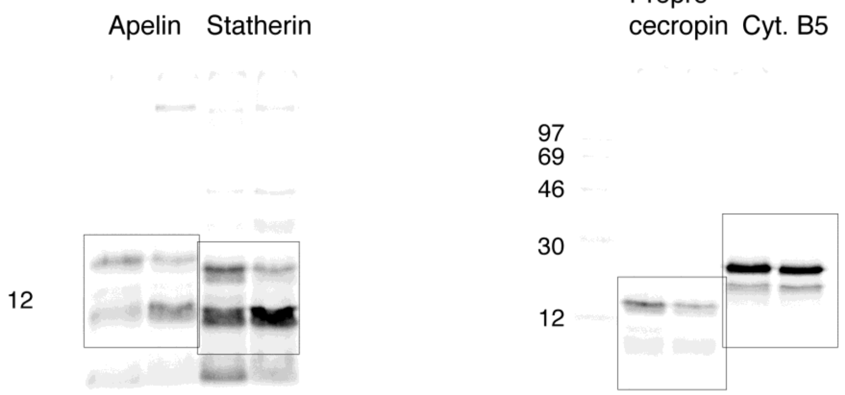

Figure 5b complete phosphorimager gel images

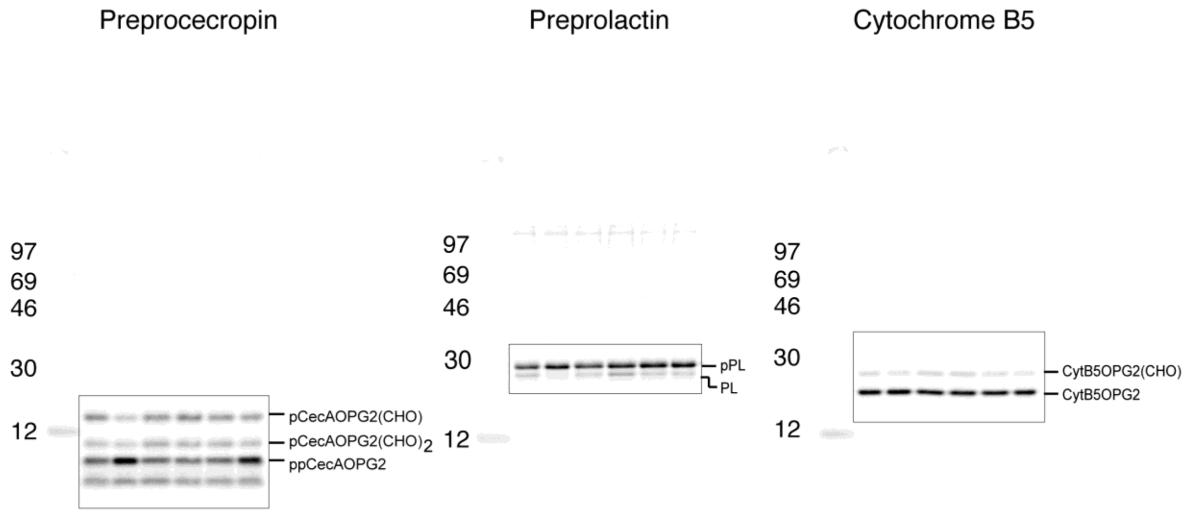

Supplementary Figure 7 Complete gel images for cropped gel panels
Regions corresponding to panels in the main figures are boxed.

Supplementary Reference

1 Sievers, F. *et al.* Fast, scalable generation of high-quality protein multiple sequence alignments using Clustal Omega. *Mol. Syst. Biol.* **7**, 539 (2011).
